# Understanding Human–Technology Relations Within Technologization and Appification of Musicality

**DOI:** 10.3389/fpsyg.2020.00416

**Published:** 2020-04-21

**Authors:** Kai Tuuri, Oskari Koskela

**Affiliations:** Department of Music, Art and Culture Studies, University of Jyväskylä, Jyväskylä, Finland

**Keywords:** music technology, technologization, 4E (embodied, embedded, enactive, and extended) cognition, human–technology relations, coevolution, appification, music education

## Abstract

In this paper, we outline a theoretical account of the relationship between technology and human musicality. An enactive and biocultural position is adopted that assumes a close coevolutionary relationship between the two. From this position, we aim at clarifying how the present and emerging technologies, becoming embedded and embodied in our lifeworld, inevitably co-constitute and transform musical practices, skills, and ways of making sense of music. Therefore, as a premise of our scrutiny, we take it as a necessity to more deeply understand the ways that humans become affiliated to the ever-changing instruments of music technology, in order to better understand the coevolutionary impact on learning and other aspects of musicality being constituted together with these instruments. This investigation is particularly motivated by the rapid and diverse development of mobile applications and their potential impact, as musical instruments, on learning and cognizing music. The term *appification* refers to enactive processes in which applications (i.e., apps) and their user interfaces, developed for various ecosystems of mobile smart technology, partake in reorganizing our ways of musical acting and thinking. On the basis of the theoretical analysis, we argue that understanding the phenomenon of the human–technology relationship, and its implications for our embodied musical minds, requires acknowledging (1) how apps contribute to conceptual constructing of musical activities, (2) how apps can be designed or utilized in a way that reinforces the epistemological continuum between embodied and abstract sense-making, and (3) how apps become merged with musical instruments.

## Introduction

Technology may be commonly understood as a human-made, tool-like resource. But in popular discourse, the development of technology is also depicted as an inevitable process that evolves independently from humans, with a nature of its own occurring outside of being a human. This sort of cultural construct affords conceptualizations of technology as an autonomous force that has an impact on people. The impact depends on whether technology is treated neutrally as a tool for some purposeful use or if it is treated as a non-neutral force that always has an effect on society regardless of its proposed uses (Savat, 2012, p. 2). In regard to education, a lot of discussion has emerged asking how technology has transformed, or will transform, the ways of teaching and learning (e.g., Gouzouasis and Bakan, 2011). In terms of musical activities, critical discussion has also been presented as to how technologies may enhance or degrade learning of “natural” musical skills, musicianship, or musical understanding (e.g., Aho, 2009; De Souza, 2017), or how they are able to transform people’s embodied relationship with music (e.g., Leman and Nijs, 2017), even constituting bodily choreographies of musical activities (Tuuri et al., 2017).

Technologization and digitalization is often treated as something that is either detrimental or advantageous to our natural human abilities. Or it is seen as something that we need to get a hold of in order to keep up with the changing world. At one end of the scale, technology may be seen as a threat to “natural” ways of being and acting human, or a power that we cannot control. And at the other end, it can be seen as a promise of a new kind of humanity, transcending the limits set by our biological bodies and fallible human intelligence. From the perspectives of non-dualistic 4E (embodied, embedded, enactive, and extended) cognition (e.g., Van der Schyff and Schiavio, 2017; Newen et al., 2018) and coevolution with technology (e.g., Ihde, 1990), we will see that the above-presented view rests on the somewhat misleading distinction between “natural humanity” and “unnatural technology.” In terms of the evolutionary continuum, technology has been an integral part of the development of humanity for so long and in so fundamental a way that it is difficult to conceive of a world, and humanity, without technology. Technology (or *technics*) thus constitutes essential characteristics of humans, and it is even possible to argue that humans “are essentially defined as prosthetic beings,” meaning that the boundaries of humans in relation to technologies are not fixed but “both plastic and vulnerable” (e.g., De Preester and Tsakiris, 2009, p. 308).

In the context of education, discussion of technology is often framed by a political rhetoric of educational reform that uses terms such as “transformation,” “radical reform,” “modernization,” and “irreversible change” (Purves, 2018, p. 144). At the same time the talk of “under-digitalized” schools depicts current educational practices in many ways as old-fashioned with respect to the utilization of modern technology (e.g., Parviainen, 2015, p. 5; Apple, 2008, p. 244). This view posits a necessity of “harnessing” the emerging power of technologization, whether it be more efficient ways of learning, more efficient or less costly ways of organizing education, or just keeping education up to date with the changing world, providing students with skills they need in a technologized society and working environments. According to [Purves (2018), p. 144], “music teachers (perhaps more so than colleagues in many other subject areas) have faced exhortations to re-equip, re-skill, and re-consider practice in the light of large-scale technological developments.” Moreover, an understanding of technology and its impact on music-making “has become vital to the success of the twenty-first-century musician” and may even be considered as a new core discipline within music education curricula (King, 2018, p. 164). With the present work, we wish to tap into this discourse about technologization of music education by highlighting the need for critically assessing the relation between humans and technology and any pre-conceived assumptions concerning it.

This paper is a theoretical investigation about the two-way relationship between humans and technology. By bringing together the theoretical approaches of enactive (or 4E) cognition and Ihde’s (1990) post-phenomenology, we aim at developing an understanding of the coevolution of technology and human musicality in relational terms and how the experience of technology and music is co-constituted in the contexts of use. Music technologies, such as musical instruments and notation techniques, have always been shaping musical practices. The first technological inventions in this area date back to the very dawn of “musicking” humanity (Ihde, 2013; Himonides, 2018). Recently, mobile devices, such as smartphones and iPads, have permeated the field of various musical uses. Our rationale is that, in order to understand musicality, or musical mind, it is necessary to better understand our coevolutionary relationship with the instruments (i.e., any music-related technologies) it adopts, that is, how the present and emerging technologies, becoming embedded and embodied in our lifeworld, inevitably constitute and transform musical practices, skills, and ways of making sense of music. Hence, musicality is inevitably constituted with these instruments. And this refers not only to a traditional conception of musical instruments but essentially to all kinds of technologies and human–technology interfaces that are potentially incorporated into musicality. With respect to music education, our aim is to provide a ground for an open-ended and reflective view of technology as a co-constitutive part of making and understanding music and also to encourage pedagogical practices that are based on “possibilities, imagination and relationality,” rather than on conformity to conventional ways of thinking (see Van der Schyff et al., 2016, p. 81).

This investigation is particularly motivated by the rapid and permeating development of “apps,” i.e., software applications designed and developed for various ecosystems of mobile technology, and their potential impact, as musical instruments, on learning and cognizing music. For users of these smart devices, apps present maybe the most prominent framework for conceptualizing new music technologies. By using the term *appification* (instead of mere technologization), we want to emphasize the pervasive transformative processes of musical practices becoming more and more extended to apps (e.g., Morris and Murray, 2018). Appification may also refer to enactive processes in which apps and their user interfaces (UIs) have an effect on both our culture and cognition by reorganizing our ways of musical acting and thinking (see Noë, 2015). Because of the rapid and constantly diversifying nature of developing new apps, compared to introducing new hardware implementations, they should well represent a reciprocal co-development of music technologies and their users.

In the following sections, we will start our inquiry by outlining the idea of appification with respect to the 4E framework, highlighting the biocultural perspective of human–technology coevolution. By building upon Ihde’s (1990) post-phenomenological account of human–technology relations, we analyze some recent discussions concerning technology in music education, focusing on (technological) reification of music (Van der Schyff, 2015), issues of cognition and corporeality in new musical practices (e.g., Aho, 2009), and different modes of mediation (Leman and Nijs, 2017). Finally, we discuss developing a better coevolutionary understanding of the human–technology relationship for the age of appified music learning and the ways to foster pedagogical thinking that emphasize deeper ecological and relational understanding of how we humans, music, and technological instruments are constantly co-constituted in the relations between each other (Verbeek, 2001).

## 4E Cognition, Biocultural Coevolution, and Appification

As noted in the *Introduction*, a considerable part of the resistance to as well as anticipation of technology can be seen as stemming from an idea of technology as something unnatural (see Hayler, 2015). Based on this premise, technology is easily seen as a kind of “insulating” layer between humans and the world, making us less “in tune” with reality, or as something that may corrode our natural human abilities and tendencies. In order to understand appification, and to consider its implications, we need a more balanced view of the relationship between humans and technology. In this paper, we follow the ideas put forth by 4E approaches to cognition and biocultural coevolution, aiming at a relational and experiential view of the mind and technology. While 4E approaches can be understood as an umbrella term for a “research program” of several different and sometimes conflicting theories, in this paper, we rely mostly on the enactive approach and consider the four Es more as referring to ways of characterizing the nature of cognition as biologically grounded and phenomenologically plausible.

A starting point for a biologically based view of cognition would be that every cognitive organism is necessarily an organism within an environment (Maturana, 1978). Cognition is not something that is pre-given but something that is dependent on the evolutionary and developmental history of living and therefore cannot be separated from the environment, supposing either environment-independent cognition or cognition-independent reality (Maturana, 1978; Maturana and Varela, 1987; Thompson, 2007). From a biological perspective, the basis of cognition is understood in relational terms as an ongoing history of structural coupling between an organism and an environment, stressing the mutual co-constitution of both, or necessarily *embedded* nature of cognition, in the process of living (Varela et al., 1993).

Another premise for a biological view of cognition is the fundamental need of living organisms to maintain themselves. Consequently, as living is not a static property but an active process, cognition can be seen as essentially an activity of creating and maintaining viable relations to the environment, which also means the creation of a cognitive domain or niche proper with respect to the needs of the organism (Maturana, 1978; Maturana and Varela, 1987; Di Paolo et al., 2014). Besides this dynamical notion of coupling or mutual adjustment between an organism and an environment, the self-maintaining, or autonomous, nature of organisms implies also that subjective experience cannot be separated from cognition. Instead, because of the need for self-maintenance, organisms have a perspective on the world through which the interaction with it is always significant and valenced (Di Paolo et al., 2014). Cognition is thus *enaction* or the active and ongoing pursuit of the organism of “bringing forth a world,” the constant process of making sense of the environment to establish meaningful (with respect to maintaining itself) relations (Varela et al., 1993).

Seeing cognition as sense-making activity of an organism in an environment implies that there is no fundamental distinction between bodily action and cognition. Instead, as organisms are bodily embedded in the environment and carry out their sense-making activities according to their bodily needs, cognition is fundamentally bodily action, that is, *embodied* (Varela et al., 1993; Johnson, 2017). These active and bodily relations to the world can be described as affordances, which refer to how an organism experiences the world and its objects as opportunities for interaction based on the bodily capabilities of the organism (Gibson, 1979; Heras-Escribano, 2019). In this sense, the act of, for example, grasping a door handle in order to open the door is already a cognitive act of a pre-conceptual and pre-linguistic form (i.e., an act of understanding the object as “graspable” based on the “resonance” between the needs and abilities of the organism and the environment).

Overall, the biological view of cognition posits it fundamentally as the activity of adaptation of an organism to the environment. This is importantly a transactive or transformative process: as there is no pre-given cognition or pre-given environment, the relation between the two is always that of co-constitution (Varela et al., 1993; Di Paolo et al., 2014). Cognition can thus also be considered as *extended*, or rather *extensive* and fundamentally world-involving (Hutto and Myin, 2017), meaning that sense-making activities may be offloaded to environmental resources, essentially widening the cognitive domain, or possibilities for action, of the organism. While the most common sense example of such is probably the use of notebooks as an extension of biological memory (Clark and Chalmers, 1998), we should also note how very basic tools, such as hammers and saws, make it possible to disclose the environment with new kinds of affordances (Hayler, 2015) and how our supposedly very basic cognitive capabilities, such as making fine-grained distinctions or thinking in terms of mental images, can be seen as based on scaffolding our cognition with language and pictures (Hutto and Myin, 2017).

In order to understand technology, such as apps, in this biological framework of cognition, we could start by stating that technologies are obviously extensions: they provide us with new ways of acting in the world, and therefore new ways of making sense of our environment (Stewart, 2014; Hayler, 2015). Because of the transactive/transformative nature of cognitive extension, technologies can be said to become also constitutive parts of cognitive systems (see Dotov et al., 2010). One may speak of *incorporation* of the tools into one’s body schema, a pre-reflective bodily experience of the world, which changes the perceived capabilities and potential for action (De Preester and Tsakiris, 2009; Thompson and Stapleton, 2009; Hayler, 2015).

This framework of co-constitutive relations has implications for understanding technology. The first of these is the often cited idea that our most basic relation to technology is in the use of them in skillful activity, or what Heidegger calls “ready-to-hand,” in contrast to the disinterested and theoretical attitude of “present-at-hand” (Ihde, 1990; Hayler, 2015). This means that we most often experience technologies as “for something” or “in order to,” being focused on the task at hand and not on the tool in itself. In accordance with the co-constitutive relation between mind and world, people become reliant on the technology and take for granted the ways of thinking, or organization, that the use of technology fosters (Noë, 2015). [Hayler (2015), p. 91] refers to this as the *domesticating* effect of technology, stressing by the choice of word the deep connection between technology and what it is to be human.

Another relevant notion is that technologies never reside in isolation but always refer to other tools and practices. This network effect of technologies is not only about other tools but also about the intrinsically social lifeworlds they belong to (e.g., Ihde, 1990). As Hayler (2015) notes, technologies are *communal*, which means that they only exist within networks of users and refiners, which play a part in structuring the interaction of the user with technology. Simply stated, we never experience technologies “as such” but in a context of social practices and ways of interacting with technology (Hayler, 2015, pp. 72–75). The communality of technology means also that learning to use technology is not only a technical but also a social process: we learn to perceive the possibilities of technological interaction (how and why to use the tool) by witnessing others actualizing such possibilities (Hayler, 2015, p. 74).

With this framework, we have attempted to bring together ideas from different theoretical backgrounds, namely, 4E cognition (understood here through an enactive approach), post-phenomenological philosophy of technology, and theory of biocultural coevolution. While these approaches indeed have their own theoretical assumptions and focuses, we consider them to be compatible enough with respect to their treatment within the argument of this paper. Most importantly, despite the differences across theoretical backgrounds applied here, the main premise of technological co-constitution of mind in action is taken to refer both to the biological idea of structural coupling between organism and environment and to the phenomenological idea of intentional co-constitution of experience. Such a fusion of conceptual approaches is in line with the original motivation of positioning the enactive theory of cognition inside a deep circulation of sciences of mind (i.e., cognitive science) and human experience (i.e., phenomenology) (Varela et al., 1993; Thompson, 2007). This is also akin to the way that Noë (2015) presents human life as being organized by skillful *activities* that permeate our biological, bodily, and cultural constitution, as well as our subjective and intersubjective experience.

Through this more general notion of co-constitution, applications are seen as a recent example of technological sense-making and experiential world-building. By becoming incorporated into our sense-making practices, applications partake in how we interact with and understand the world, a process that we have called appification. Gardner and Davis (2013) highlight how this change is not only about favoring app-based solutions (and perhaps neglecting aspects not yet accessible by apps) but also about new ways of thinking in general. As they see it, the app generation is characterized both by the use of apps and by the adoption of the “app mentality” in everyday life:

The app mentality can be considered an algorithmic way of thinking: any question or desire one has should be satisfied immediately and definitively. There is little room for ambiguity or sitting for a time with uncertainty before arriving at a decision or insight (Gardner and Davis, 2013, p. xi.).

Such an app mentality is especially linked to an increased aversion to risk and the craving for well-defined and neatly “packed” solutions in different facets of life (Gardner and Davis, 2013). We may see this as an example of how technologies potentially organize our ways of thinking and acting in a very pervasive manner.

## Technologized Musicality

The potential of the 4E framework, with its focus on bodily (inter)action and creative meaning making as the core of cognition, has been well acknowledged in the context of music. With scholars investigating different aspects of musical experience, such as musical emotions (Krueger, 2014; Schiavio et al., 2017), sociality of music (Loaiza, 2016), music education (Van der Schyff, 2015), and ontology of music (Schiavio, 2012), in the light of an active and embodied view of the mind, one may even speak of an emerging paradigm of 4E music cognition. The very basic idea of 4E approaches to music cognition can be seen as an attempt to provide an alternative to long-held cognitivist views of the mind and music: instead of seeing music and the experiencing subject as distinct entities, focusing on the mechanistic and representational information processing activity of cognitive processes, music is regarded as something that emerges from the fundamentally bodily interaction between an organism and its musical world (Schiavio, 2014; Schiavio and van der Schyff, 2018). The focus is not on the sub-personal cognitive mechanisms or on the music as objective things but on the action done with music, or on music as a verb, *musicking* (Small, 1998), instead of a noun: music as something that is done rather than something that exists in itself as an objective thing. As an example of such an approach, Krueger (2009) presents the activity of listening as “probing, exploring and manipulating both the sonic space as well as the musical components” that have a constitutive role in shaping the music (Krueger, 2009, p. 111).

This idea highlights how the experience of music is not about passive reception but about active doing/creation. Music is made sense of or constituted in the action and not something that lies outside of us (Schiavio, 2014). Sense-making, and the meaning of music as well as experience of music, is grounded in and constrained by our capabilities of bodily action. Following Schiavio (2014), we may consider the experience of music in terms of musical intentionality, as a relation between musical subject and object, which is constituted by musical affordances: our motor repertoire or vocabulary of musical acts by which we resonate with possibilities for interaction afforded by a musical object.

A crucial implication of the co-constitution of musical subject and object is that there is no “music as such,” a notion that several researchers trying to define music have accepted, but only a variety of ways for manifesting music in concrete interaction (Schiavio, 2012, 2014). As such, it is a bit questionable to talk about somehow inherent qualities of music, such as its detrimental or benign effects (or any “effects” at all) in any predetermined and one-directional sense (Sloboda, 1999), natural musical skills, or particularly “musical” meaning (see DeNora, 1986). However, this should not be understood as a claim of music’s insignificance or “meaningless” but rather as a reason to consider the way music is (made) meaningful and the properties of our biological being this meaningfulness is grounded in. One way of approaching this theme is Noë’s (2015) proposition of art as “strange tools” and its relation to what it is to be human.

The core idea in Noë’s (2015) theory is that human life is governed by habitual ways of skillful activity, or technological practices, that organize our life. Art is a way of bringing these ways of doing, our technological organization, into view and therefore allowing us to change them. As such, art is about investigating our very basic and taken-for-granted ways of living, a mode of explaining ourselves to ourselves. For example, pictures working as art disrupt our ordinary ways of looking by not allowing “functional” engagement (i.e., by being “strange” or useless tools to use for seeing and showing), thereby disclosing to us our habitual way of seeing and the pictorial economy we live in Noë (2015), pp. 165–167; see also Gallagher (2011). Going against the traditional ideas about the basis of music, Noë argues that music is not about sounds any more than discussion is about sounds we make to each other (Noë, 2015, pp. 184–187). Instead, listening to music is oriented toward the practical activities of doing music and therefore to the ways we are rhythmically, tonally, and melodically organized. In this way, music is about investigating our more pervasive “musical” organization, how we may, for example, make sense of things in terms of harmony or rhythm (Noë, 2015, p. 188).

Noë’s (2015) view would frame the essence of music as an activity, in line with the ideas of musicking that is deeply connected to our more general “musical” being-in-the-world. What follows from this is that the ways we understand music become entangled with tools and technological practices we use in our musical activities. Mobile apps have already permeated into these practices that constitute our musical worlds, thus organizing and reorganizing our musicality in a process of appification. One may consider, for example, how mobile music listening apps, by providing personalized and context-aware access to online streaming media (see Wang et al., 2012), co-constitute our ways of listening to and thinking about music. The main point here is that the idea of “music in itself” or talk about some “essentially musical” features tends to conceal the actual (technological) practices and diverse meanings of musicking. This highlights how, when considering music cognition or music education, we should not be tied to pre-conceived notions of music but rather investigate the ways music is made meaningful in the act of musicking (see, e.g., Schiavio, 2012).

Although Noë talks about technology in a more general sense as (*technç*), referring primarily to skillful activity alongside of material tools, the basic idea is that music, as a way of doing, is something that we construct, and that constructs us, according to the practices we have at our use. In this view, music (as we understand it within our modern context) is technological practice from the very beginning (Tomlinson, 2015; see also Ihde, 2013). However, this technologicality of music doesn’t refer only to instruments. Within the 4E view, there are no fundamental differences between the concrete practices, the action, and what “things are” experientially. This means essentially that our musical understanding, musical sense-making, “what music is,” is tied to the technologies through which we create our musical worlds.

## Reification of Music Through Apps

Based on the embodied and enactive perspective on human cognition, which frames human musicality basically as an interactive and relational activity, Van der Schyff (2015) introduces the notion of *reification* to the theoretical discourse of music education. More precisely, he discusses reified ways of understanding music through conceptual categories or other objectifications of musical phenomena that may provide a reductive illusion of musical reality as being inherently constituted upon them. Maybe one of the strongest examples of this phenomenon can be seen in the Pythagorean definition of musical intervals through numerical abstraction and its pervasive influence on even today’s understanding of music as inherently mathematical organization, or at least, highlighting the objective “purity” of musical scales as the very basic building blocks of music (e.g., Parncutt and Hair, 2018; Knakkergaard, 2019). Reified conceptions of music presumably are connected to and involved in the construction of broader cultural ideologies, such as those relating to the historical canonization of Western music (and its superiority), tendencies of commodifying music as pleasure technology (Pinker, 2009) or “pharmaceutical” utility (see Sloboda, 1999), as well as ideologies emphasizing the power of mathematics and natural sciences in formulating music theory (e.g., Derkert, 2007). Most importantly, however, reification in general exemplifies a dualistic view of defining music as a prescribed organization of the “outside” world. This type of view overlooks the possibility of enactive structuring of music and its meanings in the history of embodied, world-constituting interaction with the environment. In all, the motivation of van der Schyff’s argument is in emphasizing the importance of critically reflective, “life-based,” and ontological approaches to education, promoting a critical attitude toward “evident truths” and fostering the curiosity of the learner for engaging with his/her musicality in developing a deeper experiential and ecological personal understanding of it.

Throughout history, new technologies applied to musical practices have been involved in creating reified views on music. Already in the era of early musical notation, Aristoxenus, a well-known music scholar of the 4th century B.C.E., criticized notation because it only tells the size of intervals but ignores their functions in melodic formulation (Mathiesen, 1999). In other words, notation techniques promote the focus on the concept of the musical note (i.e., a chunk of melodic realization), reifying it as an essential object of music-making. Aristoxenus saw that “notation may make it easier for amateurs to see something of the nature of music” (*ibid*, p. 323), but the significance of intervals and notes as a part of a melodic continuum remains unseen. In contrast to the Pythagorean approach, Aristoxenus highlighted the primacy of lived experience and perception of music in his theorizations. This is demonstrated in the ways he defines melody fundamentally as *motion* of voice that involves processes of stretching and relaxing (*ibid*, p. 303–304) and notes merely as *positions* of certain pitch, on which the voice falls (*ibid*, p. 306). Musical instruments (such as the lyra and aulos) also embody discrete notes (as affordances to a musician). Furthermore, Aristoxenus did not consider the musical instrument an embodiment of musical nature but, rather, a medium for expressing that nature “under the control of the senses” (*ibid*, p. 323–324).

Technologies such as musical notation systems or musical instruments embody and exhibit a certain conceptual construction of “how music is organized” and “what I can do with it,” while at the same time, they might hide other ways of conceiving and imagining musical possibilities. We may see this phenomenon as comparable to *the law of instrument*, introduced by Kaplan (1964) in his discussion on scientific methods and techniques. His famous description of the law goes as follows: “Give a small boy a hammer, and he will find that everything he encounters needs pounding” (Kaplan, 1964, p. 28). According to this idea, an instrument that is familiar to its user – such as a musical instrument and the organized activities (i.e., playing techniques) it embodies – produces a cognitive bias to see everything through the possible use of the instrument. For a trained pianist, the piano and its keyboard likely co-constitutes a “world” of musical possibilities, while the given instrumental skill also produces a “trained incapacity” (*ibid*, p. 29) to think of music in a different way. In terms of reification, we might expect that a somewhat similar bias toward understanding music through the piano keyboard could also apply to non-trained pianists, due to the pervasive role of the piano and its keyboard as an educational instrument in the Western culture. But, besides being a part of a concrete instrument, the piano keyboard has arguably also become a conceptual construction and a culturally shared model for understanding musical organization. According to the study of Kell and Wanderley (2013), it also appears to be the most popular UI concept in musical apps.

The form factor of an instrument, or the UI design and functionality of a smart device app, inevitably endorses certain orientations of playing techniques, as well as conceptual formulations of “musical order.” Mostly this should be considered a natural coevolutive aspect in the developmental continuum of music technology, but it might be seen as problematic in cases where ignorance^1^ of musical discovery, beyond the frame of the instrumentally reified (possibly superficial) model of musicking, gets promoted. The approach of *critical ontology* toward music education (Van der Schyff et al., 2016) strongly encourages acquiring an ecologically deep, experience-based understanding of music and the musical self in relation to technologies. [Kaplan (1964), p. 29] provides a guideline to avoid the deficiencies of a “trained incapacity,” by appreciating “the greatest possible range of techniques” in training. Similarly, achieving the goals of ontological education should require an appreciation of the plurality of possible or imaginable instrumental^2^ paradigms and techniques of musical interaction and thinking. In regard to apps, it should be noted that these instrumental paradigms and techniques not only concern apps that behave like traditional musical instruments. Music production apps represent another important genre of musical applications (which will get more attention in the next section of this paper). These types of apps also incorporate conceptual paradigms that reify musical understanding. For example, within their editing interfaces, musical elements are usually visually objectified and sequentially positioned in a timeline or a grid, thus promoting a given conceptual idea of how music is built.

Music apps for today’s touch screen devices can potentially utilize various musical interaction patterns (see, e.g., Flores et al., 2010) and different ways of organizing musical elements within them. These design options do not need to follow the typical “a button triggers a note” paradigm, but rather, designers are able to freely re-imagine ways of playing, that is, how finger movements and gestures are mapped to control the sound output (see, e.g., Levitin et al., 2002; Miranda and Wanderley, 2006). Contemporary smart devices offer a relatively unified technological framework for designers to work with: for example, the constant form factor of the basic device (such as a smartphone or iPad), a capacitive touch screen that can handle data input from multiple fingers, motion sensors, a microphone, networking abilities, and enough processing power for real-time sound analysis, synthesis, and even machine learning (see, e.g., Essl and Lee, 2017). Within this framework, designers of music apps can opt for very different approaches to utilizing these resources in manifestations of musical UIs. For example, Kim and Yeo (2012) introduced a collaborative mobile music performance system that is based on the mobile phone’s digital compass data for tracking inter-performer interactions within an ensemble. Different mappings of sound control can fuse together into a single interaction pattern. For example, the interface of the iPad app *Orphion* (Trump and Bullock, 2014) consists of visual circles that basically function like buttons or pads, but in addition to a mere triggering of a sound, different ways of touching the pads and after-touch gestures incorporate control of different expressive parameters. Orphion’s interface also demonstrates versatility in providing different layouts for different musical genres and playing situations, with different morphological arrangements of pads varying in number, size, position, and the mapping of notes. Alternative arrangements for musical input may be required for the sake of ergonomy, creativity, or physical disabilities of a player. More generally, in respect to reification, such a multifaceted approach in UI morphology presumably yields less rigid conceptual constructs on musicking and musical understanding, by demonstrating the possible ways of organizing musical interaction instead of adhering to an axiomatic arrangement.

Creative experimentation with different interface paradigms for musical interaction is potentially a very fruitful endeavor, as can be seen in the light of Baily’s (2008) account of the physical relationship between a musician and an instrument:

A musical instrument is a type of transducer, converting patterns of body movement into patterns of sound. The technical problems that arise in learning to perform are likely to be very revealing about music and the human body, with what goes on at the human/musical instrument interface, with “ergonomics” of the music, showing how it fits the human sensorimotor system and the instrument’s morphology (Baily, 2008, pp. 123–124).

But it is not just issues of physical interaction that are potentially being elicited in the processes of experimenting with different music app interaction designs. Different UI morphologies also partake in cognitive processes of musical conceptualization and thus co-constitute a creation of musical understanding. It is possible to identify such structures of musical understanding and make them visible in the design process (Wilkie et al., 2010). Hence, while manifesting a certain conceptual construction of music, new music apps are also able to create and transform them. For a music educator, this can be considered both a challenge (of supporting reified views on music) and an opportunity to empower learners to explore and discover their personal/embodied relationship and ways to work with music. In order to provide opportunities for this kind of tangible exploration, teachers may consider making do-it-yourself musical interfaces together with students. For example, currently available Arduino-based microcontrollers make it possible to easily implement and modify capacitive touch sensors (by utilizing everyday objects such as sheets of paper) for experimental testing and demonstrating of different designs (see, e.g., De La Cruz and Bhatia, 2018; Hughes, 2018; see also Giraud and Jouffairs, 2016). Through this sort of maker pedagogy approach (see Bullock and Sator, 2015), learners may actualize a certain “hacker mentality” (within the limits of the given maker environment) by being able to make modifications to morphological arrangements of the UI. This affords the learners an opportunity to critically reflect on different techniques of musical interaction and deconstruct axiomatic design patterns for the purpose of fostering new conceptual knowledge. In this way, different ideologies and conceptual approaches – thus potentially reifying aspects – inherent in musical interfaces could be made experientially explicit to the learners.

## Craftsmanship in Digital Musicking

As we can see from the above discussion, technologization and appification of musical practices – while transforming the tools of music-making – are able to promote certain conceptual attitudes, values and ideologies involved with these practices. In this section, we will briefly discuss how musicianship evolves with these developments, and in particular, how the “craft” of music, referring to the ways of creating and doing music, is being transformed in relation to the human body. Let us start with a rather extremist approach taken by Thibeault (2018), in which he frames the present music education with the concept *postperformance world*, where “performance is sometimes an option but often impossibility, and rarely the avenue by which we experience music” (*ibid*, p. 204). It is certainly true that musical experiences are often achieved from recordings rather than live performances and that music production practices have receded from performance; thus, music is being *constructed* rather than directly originating from the body or the instruments of a player (Ihde, 2013; Thibeault, 2018). In contrast to Thibeault’s argument, however, this is not to say that the role of musical performance would be totally omitted or even significantly reduced in musical experiences. According to Ihde (2013), new music production practices rather exemplify a different type of player–instrument (i.e., embodiment) relation in the involved performance. In such practices, the composer/editor in a way becomes a player as well, by “playing” the music production programs, apps and electronic devices, and finally fixing the constructed result as a “recording” (Ihde, 2013). Actually, it is common that enthusiastic YouTube authors (such as Doctor Mix)^3^ may explicitly show in their videos how a certain classic song has been constructed, thus effectively deconstructing and re-enacting these new performative practices of music-making. Of course, as we can observe from the videos, performance is manifested differently here when compared to the classic player–instrument relation. For instance, playing music on a keyboard appears to be more like a way of inputting sequences of symbolic data into the computer system, in which it is edited and treated as patterns and building blocks of music in so-called non-linear editing environments (see, e.g., Rumsey, 2003).

In her article, Simon (2018) has analyzed a music app, *iMaschine 2* (by Native Instruments)^4^, and argues how this software promotes an ideology of app culture that defines musical creativity by production efficiency and outcome rather than by process. According to her analysis, the app is basically a hybrid between music software and a productivity tool that in its interface design manifests values that “infiltrate the music composition process and esthetic outcome as well as the marketing discourse that encircles the app” (*ibid*, p. 268). The workflow of composing music in iMaschine 2 is based on constructing music in short patterns. While it affords the user to play music (online) with sample triggering and keyboard interfaces (i.e., app as an instrument), the overall performativity is biased toward off-line editing and composing of the musical result (i.e., app as a productivity tool). In this sense, the app seems to correspond well with the new kind of player–instrument relation discussed above. This notion also seems to be in line with the survey results of Tanaka et al. (2012), where “sequencing with real-time control of parameters” (i.e., productivity tool bias) was identified as “one of the most common modes of musical interaction” with mobile music apps.

Simon (2018) in particular criticizes the ideology of app culture for “fostering the feeling of accomplishment without the blunders and “mistakes” that would otherwise attend music production” (p. 268). The prevention of errors, such as playing in a wrong key, thus reduces the possibility to discover and create through failure. In general, the ethos of making the production easier and more efficient (e.g., maximizing the value of a minimal finger sweep) contributes to the trend of extending/translating activities and skills to human–technology assemblages that once resided in the domain of human labor, acquired through bodily engagement (see Fuller and Goffey, 2012; Parviainen et al., 2013). In regard to new musical instruments and music production tools, this suggests that the action–sound relationships in music-making (i.e., what kind of physical actions and techniques are used to make sounds) may become estranged from our habituated bodily knowledge of action–sound couplings (see Jensenius, 2013).

As way of summarizing this discussion, we can outline arguments about an ideological trend in digital musicking that promotes the constructed result over performance. But does this also mean that corporeality is reduced, and musicking becomes less tactile? At least, Marko Aho has argued that “the user (of digital tools) is often a programmer or a composer working solely with his or her cognition, rather than a tactile player” (Aho, 2009, p. 25). This implies the existence of another, consequential trend in musical craftsmanship of the digital age that emphasizes cognition over embodiment. Aho (2009, 2016) points out that the instrument itself is a lifeless object – a technological resource – which comes to life only by the player’s body movements. These movements, in tandem with the player’s tactile and kinesthetic senses, inevitably constitute a style of playing, which is a physiological and expressive resource that enables infinite variation of how sounds are created. In Aho’s argument, such interplay with the instrument is essentially based on bodily feelings rather than cognition. With digital instruments and interfaces, the relationship between the style and the resulting sound is essentially parametric (see, e.g., Hunt et al., 2003), meaning that the player’s body movements must be transformed into numeric data, as an input to the system. The data represent these movements in a selected dimension or category. As a consequence, the mapping of the playing style and the resulting sound is arbitrary and finite, and ultimately bound up with the parameters, which are preset by the design and not a result of an enactive negotiation between the player and an instrument. The parametric organization of the musical data also endorses cognitive and analytic work in music-making. In [Aho’s (2009), pp. 25–31] analysis of these issues, musicianship becomes literally *handicapped* by the loss of “transcendence through flaws” and the loss of “finding the instrument” with your body, as he puts it. Of course, the intention behind these arguments is not to demonize digitalization of music-making but, rather, to bring up these questions of tactility and cognition in relation to music instrument performance.

## Modes of Technological Mediation

To reiterate the basic approach of this paper, we have aimed to consider technology in a relational framework. The focus has thus been on how users experience technologies in terms of their sense-making abilities: how the experience of technology is constituted in the intentional relation. One popular way of framing this relation is in terms of mediation, in which technology is seen as modulating the user’s experience of the world (Ihde, 1990). However, the framework of 4E cognition and biocultural coevolution has certain implications for what is meant by mediation. Therefore, in this article, we consider one view of technological mediation within music education based on a different framework and aim to frame an alternative view highlighting the idea of co-constitution crucial to the 4E approach.

In a recent article, Leman and Nijs (2017) discuss the effectiveness of technology in music education, framed as the question of whether technology enhances or degrades instrumental music learning. They propose that the answer to this should begin by considering the embodied cognitive architecture at play in music learning and its compatibility with education technology. Leman and Nijs (2017) begin by offering a definition of technology. They rely on a fundamental idea of technology as an extension of natural human capabilities, making a distinction between music *mediators* that “allow the realization of natural-born abilities for music-making” (e.g., musical instruments) and music *facilitators* that “make such a realization easier” (e.g., educative technologies). These two types of technology with their different roles make up the “technology-based mediation–facilitation framework,” the alignment of which to cognitive architecture they are investigating. [Leman and Nijs (2017), pp. 24–25] point especially to the crucial compatibility between body and technology, which they discuss with modes of mediation.

Leman and Nijs (2017) present two different modes of mediation, or ways of experiencing technology. The first of these is the prosthesis mode, which “occurs when technology is experienced as a natural extension of the human body, such that technology becomes a part of the human body.” The second one is the dialogue mode that “occurs when technology is experienced as part of the environment, such that it acts as a device that necessitates a dialogue” (Leman and Nijs, 2017, p. 25). As they see it, the goal for prosthesis-like technology is the transparency of the tool, which we referred to as incorporation, which allows for unhindered flow from musical ideas through instrument to musical output that has a possibility to affect a listener in a way desired by the musician. To this end, music education aims at aligning the cognitive architecture with the technology, that is, training of the schema-based and fine-motoric capabilities in order for the musician to produce the wanted sensory outcome. While this transparency is especially the aim for prosthesis-like mediation, most obviously musical instruments, [Leman and Nijs (2017), p. 26] stress also the need for similar compatibility between technology and human cognition for effective dialogue-like mediation.

Following this line of thought, effective mediation is about proper alignment of technology to human cognitive architecture, which serves the goal of “transparent” transmission of musical ideas into desired sensory outcomes. As [Leman and Nijs (2017), p. 32] suggest, this can be linked to an embodied-constructivist approach, in which the compatibility of the technology with the user’s sensorimotor capabilities serves efficient knowledge construction. To contrast this constructivist approach with a 4E view of mediation, the most crucial difference is the focus laid on the co-constitutive nature of interactions in the latter (see Li et al., 2010). As such, technological mediation is not that much about “transference” of ideas or action through technology to the world but about mutual construction of those ideas and actions in the relation between the human and technology (see Verbeek, 2015).

As we discussed with respect to 4E approaches to music cognition, it is a bit problematic to propose a clear-cut distinction between natural music abilities and technologically/culturally constructed ones. Instead, we could see musical skills and ideas as emerging from the interplay of the human, technologies, and the environment, or from “hybrid extended cognitive systems” (Schiavio and van der Schyff, 2018). This is most obviously the case with musical instruments and the acquisition of instrumental technique associated with them, which, as Jonathan De Souza has noted, “affects the ways that players perceive, understand, and imagine music” (De Souza, 2017, p. 2). We might consider, for example, how the musical skills and intentions of a guitar player are shaped by the affordances of the instrument, the understanding of the player about what is possible to do with the instrument. However, we should not limit this idea to instruments but, rather, consider the incorporation of new technologies into this already “hybrid music system” as a reorganization of musical skills and intentions, such as when notation might be seen as a part of a pianist’s skill to perform the piece, or when an array of effect pedals is seen as a part of a guitarist’s expressive thinking.

The idea of co-constitutive technological mediation can be extended from musical skills and intentions to our understanding of musicking or what we consider as music (see Born, 2005). In this sense, different kinds of technological mediations might blur the distinction between music mediators and music facilitators proposed by Leman and Nijs (2017), as in the case of the previous example about the adoption of notation technologies not only as a tool for learning but also as an extension of musical abilities. Music learning video games, by having a dual role as a game and an educational application, can be taken as being especially prone to restructuring our understanding of music and musical activities. In his discussion of *Rocksmith*, a musical video game where one plays with a real guitar along with the notation scrolling on the screen, O’Meara (2016) points out how the game has a role in shaping the player’s experience and conceptualization of music. He considers especially the impact of the game’s difficulty system on the player’s perception of the songs: how what is presented as easier is deemed as essential and how this creates distinctions between the structure and ornaments of the song. Besides having a role in structuring the understanding of music, *Rocksmith* has been presented as a pedagogical tool that may provide a new kind of understanding about “serious” and “playful” aspects of music education, thereby paving the way for a “more ludic culture of instrumental pedagogy” (Havre et al., 2019, p. 29). Moreover, it could also be argued that musical video games, as well as other interactive music applications, also shape the idea of musicking. As O’Meara (2016) notes, it is not uncommon for musical video games to give rise to an online community centered on the game, nor for the players to share their performances on YouTube. In this sense, *Rocksmith* and musical video games alike may be said to participate in the creation of new musical practices and cultures.

In all, technologies can indeed be seen as mediators between the human and the world, but, as they always transform the experience of the world, they should not be seen as neutral ones (Ihde, 1990, p. 49). In line with the non-dualistic and relational approach of 4E cognition, technological mediation could be considered a relation that constitutes both the human and the world (Verbeek, 2001, 2015). Different musical technologies, instruments and musical applications included, afford (in relation to the user) different kind of action, make different kinds of musical skills and ideas possible, and partake in the creation of different musical realities. Therefore, the question of music education technology is not only about its effectiveness with respect to a given idea of music but also about musical worlds that can and should be brought forth with them. That said, it can still be argued, as Leman and Nijs (2017) suggest, that technologies incorporated as prosthesis-like extensions, by being taken as parts of the very basic sensorimotor sense-making capabilities, have less of a role in determining our constitution of the world than technologies we interact with in a dialogical manner. However, we should still remember the already technologized nature of our musical worlds and strive for a balance between fine-tuning the adaptation into particular technologies (e.g., mastering the instrument and the particular access to the musical world it makes possible) and a more reflective or experimental attitude toward the technology and the ways it shapes the understanding of music (see Leman and Nijs, 2017, p. 26). Moreover, with the new kinds of musical technologies and new kinds of musical practices they provide, we should also consider other kinds of possible relations to technology besides the two presented by Leman and Nijs.

## Music Technology and Human–Technology Relations

Similarly to the concept of music, the talk of technology in a general sense tends to conceal the fact that instead of the supposed “essence” of music or technology, we are talking about particular interactions. As the core argument of the paper goes, technology does change us, but it doesn’t change us “in itself” or in any predetermined manner. Rather, it changes us because we couple to particular technologies by concretely using them in particular situations, therefore learning new ways of adapting or relating to the world. As we may only have the world we bring forth by our action, the technologies we use for this process also contribute to this world-making.

To gain a more detailed understanding of how technologies may enter our life, we turn to Ihde’s (1990) account of human–technology relations. The account’s basic idea is to consider the interrelations of humans and the world in the case of technological interactions, or “what form of world-disclosure is *made possible by* technological artifacts” (Verbeek, 2001, p. 123). Ihde outlines four types of relations to technology.

The first two types of relations Ihde (1990) discusses are both forms of technological mediation in which we do not experience the world “directly” but by/via technological artifacts^5^. Within these kinds of mediating relations, Ihde further distinguishes two types of mediation, the first of which he calls *embodiment relations*. These refer to taking technology into one’s experience, thereby transforming the perceptual-bodily experience of the world (Ihde, 1990, p. 72). This is the relation of experiencing the world through tools, which we have referred to as incorporation (De Preester and Tsakiris, 2009; Thompson and Stapleton, 2009; Hayler, 2015) and the prosthesis mode (Leman and Nijs, 2017). The most important aspect of such a relation is the transparency of the technology: the technology withdraws from the experience to allow the experience to be of the world given through it. Another kind of mediating relation we may have to technologies is what [Ihde (1990), p. 80] calls *hermeneutic relation*. This means that technology is experienced as a representation of something, which requires reading or interpretation in order to be understood. In this way, the world is not experienced through the technology but by means of it, the prime example of which is how we experience “the world of text” through writing. With respect to transparency, hermeneutical relations are about *hermeneutic transparency* instead of perceptual transparency: while the text or musical notation is the perceptual focus of experience, it also withdraws from our experience to disclose what it refers to [Ihde (1990), p. 82].

In the case of musical technology, the typical way of considering a player’s relation to a musical instrument would be the embodiment relation, in which the player has learned to “symbiotically” embody the instrument (Ihde, 1990, p. 95). On the other hand, music technology that relies on representations of sounds, such as notational systems or visual organizing of music into blocks, would be experienced as a hermeneutic relation. However, Ihde (1990) highlights that these relations should be seen as occurring within a continuum: we can imagine situations where, for example, a conflict between expressive possibilities of the instrument and our bodily understanding of action–sound couplings (Jensenius, 2013) causes the embodiment relation to break down into a hermeneutic-like experience of “translating” musical ideas into the operation of the instrument, or when the UI of the music app exploits more compatible action–sound relations, hence turning the app into a bodily extension of the musician.

Besides these two mediating relations, the third type of relation to technology concerns the cases where the world is not experienced as mediated by technology, but rather, the technology is present “in itself.” [Ihde (1990), p. 97] calls these *alterity relations*, which refer to the experience of technology as an “other,” and the relation is in the form of “to” or “with” a technology. Examples of these relations are the anthropomorphization of tools, as when we project human qualities onto, for example, computers that seem to respond to us in an intelligent manner or when we entertain similar feelings toward technologies that we would entertain toward other people, such as when “caring for” a precious instrument. This “quasi-otherness” (Ihde, 1990, p. 100) highlights the way we may experience technology as having an autonomy that allows us to interact with it: similarly to how technology shows its “material will” in dialogue mode (Leman and Nijs, 2017, p. 26), alterity relations have a certain unpredictable character that may be experienced as frustrating (as when learning a new instrument) or as inviting exploration.

The fourth type of technological relation is the *background relation*, where technologies shape our relation to reality but themselves remain in the background (Ihde, 1990, p. 108). In this way, we don’t consciously experience the presence of technology even though it impacts on the context and environment of our experience. With respect to music technology, we may consider several technologies, such as listening equipment or technologies related to the production and distribution of music, that are absently present in our everyday encounters with music. It could be claimed that because of this absent presence, these technologies tend to have a subtle but important effect in shaping the “gestalt” of our experience (Ihde, 1990, p. 112).

While we have here considered different relations with examples of particular technologies, it should be noted that, in a co-constitutive manner, it is our relation, how we use the technology in a particular context, that makes technology what it is. As such, these relations describe different continuums along which we may experience technologies: as being an embodied or representational mediator between us and the world, being more or less present as a piece of equipment “as itself,” and being in the foreground or absently in the background of our experience of the world. At the same time, these relations describe how technologies shape our access to the world, what kind of possibilities for technological world-building they provide.

## Concluding Statements

In this paper, we have outlined new theoretical formulations for understanding the coevolution of technology and human musicality. This theoretical contribution concerns merging the post-phenomenological discussion of the human–technology relationship with the recent theoretical developments in cognitive sciences, namely in regard to enactive and 4E cognition. On the basis of the theoretical analysis and discussion, we argue that understanding the phenomenon of the human–technology relationship (in the age of mobile apps), and its implications for our embodied musical minds, requires acknowledging at least the following facets of appification:

(1) How apps contribute to constructing reified ideologies and conceptual schemes in relation to music-making.
(2) How apps can be mindfully designed or utilized in a way that reinforces the epistemological continuum between embodied and abstract sense-making.
(3) How apps become merged with musical instruments and how musical skills become merged with apps.

The first item corresponds with our discussion around reification. As has already been stated, through their UI, apps manifest a conceptual construction of music, but they are also able to transform such constructions. As a pedagogical strategy, music educators can make reifying aspects of appification explicit to learners and try to consciously select different apps for displaying different ideologies and conceptual approaches. Personal discoveries of this kind may produce empowering effects for learners, especially if combined with (conceptual) design exercises of their own music apps and musical interfaces.

The second item points out the concerns of potentially negative trends in digital musicking, namely, the outlined trends of promoting *music as construction* over process or performance and emphasizing *abstract cogito* over bodily, tacit knowledge. We would encourage music educators not to interpret these as fatalistic and inevitable negative consequences of technologization. On the contrary, they can be reassessed in terms of naturally co-constitutive changes in our embodiment relations with emerging instruments and tools of music-making. These changes can also comprise whole new types of creative agency and performative practices.

If we consider musical instruments to be defined as “transducers that convert patterns of movement into patterns of sound” (Baily, 2008, pp. 123–124), designing of new musical instruments can as well be conceived the other way around. Indeed, action–sound relationships of musical interactions can also be used to deliberately persuade the player into making movement (see Bergsland and Wechsler, 2015), thus effectively generating bodily choreographies for playing (Tuuri et al., 2017). The designers of music apps have the opportunity to redefine the role of bodily activity and performativity of music-making. Hence, the principle of “maximizing the value of a minimal finger sweep” represents only one end of the potential continuum. Collaborative music apps are also becoming more common (e.g., Essl and Lee, 2017) and have the potential to project embodied ways of interpersonal interaction into these emerging performative music-making practices. Moreover, present-day social-media applications, such as YouTube, offer examples of how app culture in general can promote new ways of musical performativity, such as in the form of people posting videos where they present their own cover versions of famous songs^6^.

The final item asks us to reconsider the relationship between apps and instruments and to see musical skills as being extended to human–technology assemblages. The merging of apps and instruments can occur in various ways. Firstly, an app can transform its end device (such as a smartphone or an iPad) into a musical instrument (e.g., Jones, 2013). Secondly, a musical instrument (such as a piano keyboard) can be transformed into a UI widget (see Kell and Wanderley, 2013) that may reside, for example, within a music production app (such as iMachine 2). Thirdly, apps can be used to augment the capabilities of physical instruments (see, e.g., Overholt, 2011). But in regard to the skills of playing a musical instrument, what could be the implications of these skills developing in hybridization with app-driven technologies? In general, this at least implies a reassessment of musical skills that “can be seen as a welcome democratic consequence of musical instrument digitalization” (Aho, 2009, p. 25). But isn’t there a risk that the changeable nature of apps could break up any developed skill relations between players and these instrument hybrids? We can at least speculate that, in terms of modes of mediation (Leman and Nijs, 2017), even small inconsistencies or variations in UI functionality (as a result of, e.g., artificial intelligence algorithms or app version updates) can potentially disrupt the emerging prosthetic relationship with the instrument and bring the player back into a more dialogical relationship with the device. On the other hand, designers can opt to utilize the existing prosthetic relations that people have with technology (such as the well-established habituation of using a touch screen smartphone with a thumb) to create a novel design framework for playing music (such as in the app *Thumb Jam*, where one can play music with a similar vocabulary of movements)^7^.

From the stances of the four Es and biocultural coevolution, neither the world, the mind, nor any technology is pre-given, but rather, co-constituted in the relations between each other (Ihde, 1990; Verbeek, 2001). This constitution is an ongoing process, and therefore, the world is always a “world-in-the-making,” which is fundamentally about the bodily activity of an autonomic organism to maintain its identity (Di Paolo et al., 2014). Conceptualizing humans and technology as co-constitutional, relational entities promises to be an appropriate way to understand how technology is reciprocally partaking in our world-making processes. From this point of view, our musical world-making is inevitably instrumental, in the sense that music technologies are incorporated into our ways with music as an organized activity (Noë, 2015). The more our musical practices entangle with apps, the more our musical worlds and musical ways become appified. The reason why mobile apps may have an even bigger transformative role than the more traditional applications of personal computing lies in the ubiquitous role that mobile technologies are taking in all kinds of everyday activities. Therefore, apps will probably have an increasingly significant role in human–technology constituted world-building.

If we accept the premise that cognition is not something pre-given but a result of the ongoing developmental and evolutionary history of successful interaction, and consider us humans essentially as tool users, we may speak of the coevolution of the human and technology, or what Stiegler (1998) refers to as technogenesis. Indeed, as several scholars have pointed out, what we consider as “human” has been shaped by technology to an extent that we could consider our ability to be molded by the tools we use as a defining trait of humanity (Hayler, 2015; Noë, 2015).

## Author Contributions

All authors listed have made a substantial, direct and intellectual contribution to the work, and approved it for publication.

## Conflict of Interest

The authors declare that the research was conducted in the absence of any commercial or financial relationships that could be construed as a potential conflict of interest.

## Footnotes

1 A similar type of epistemological masking has been discussed in the context of augmented reality (AR) applications: while providing access to a specified type of information in the environment, AR devices potentially promote ignorance in their users—denoting a condition of *not being aware of what they don’t know* (Parviainen, 2017).

2 The word “instrumental” here should be understood in the broadest possible sense, including not only conventional musical instruments and material technologies but also conceptual tools (such as notation) that entangle with musical activities.

3 See, for example, Doctor Mix (2019), deconstructing Darude’s “Sandstorm”. https://www.youtube.com/watch?v=hhEnA90PoyE. Accessed November 25, 2019.

4 See https://www.native-instruments.com/en/products/maschine/maschine-for-ios/imaschine-2/whats-new/. Accessed November 25, 2019.

5 “Directly” in this case doesn’t mean a sort of “pure” experience of the world but, rather, an experience that isn’t constituted by technological artifacts. As we will discuss later, and as we have already discussed with respect to “world-building” based on the particular perspective of the organism, the phenomenological approach of Ihde doesn’t suppose that we could ever experience the world “as such.”

6 See the YouTube user Bwana playing “iPhone Ocarina: Song of Storms Redux – SSBB Version” https://www.youtube.com/watch?v=fM9vrM-DO5k. Accessed November 28, 2019.

7 See, https://thumbjam.com. Accessed November 29, 2019.
